# Mining of potential drug targets through the identification of essential and analogous enzymes in the genomes of pathogens of *Glycine max*, *Zea mays* and *Solanum lycopersicum*

**DOI:** 10.1371/journal.pone.0197511

**Published:** 2018-05-25

**Authors:** Rangeline Azevedo da Silva, Leandro de Mattos Pereira, Melise Chaves Silveira, Rodrigo Jardim, Antonio Basilio de Miranda

**Affiliations:** 1 Instituto Oswaldo Cruz, Fundação Oswaldo Cruz, Rio de Janeiro, Rio de Janeiro, Brazil; 2 Pontifícia Universidade Católica do Rio Grande do Sul, Porto Alegre, Rio Grande do Sul, Brazil; College of Agricultural Sciences, UNITED STATES

## Abstract

Pesticides are one of the most widely used pest and disease control measures in plant crops and their indiscriminate use poses a direct risk to the health of populations and environment around the world. As a result, there is a great need for the development of new, less toxic molecules to be employed against plant pathogens. In this work, we employed an *in silico* approach to study the genes coding for enzymes of the genomes of three commercially important plants, soybean (*Glycine max*), tomato (*Solanum lycopersicum*) and corn (*Zea mays*), as well as 15 plant pathogens (4 bacteria and 11 fungi), focusing on revealing a set of essential and non-homologous isofunctional enzymes (NISEs) that could be prioritized as drug targets. By combining sequence and structural data, we obtained an initial set of 568 cases of analogy, of which 97 were validated and further refined, revealing a subset of 29 essential enzymatic activities with a total of 119 different structural forms, most belonging to central metabolic routes, including the carbohydrate metabolism, the metabolism of amino acids, among others. Further, another subset of 26 enzymatic activities possess a tertiary structure specific for the pathogen, not present in plants, men and *Apis mellifera*, which may be of importance for the development of specific enzymatic inhibitors against plant diseases that are less harmful to humans and the environment.

## Introduction

One of the major challenges for plant breeders is to maintain high levels of quality and production of cultures. Diseases caused by plant pathogens are one of the main factors limiting the productivity of large commodities, such as soybean (*Glycine max*), corn (*Zea mays*) and tomato (*Solanum lycopersicum*) [1,2]. Use of pesticides is one of the most commonly used alternatives to plant pathogens control, being used in a wide variety of crops [3].

Pesticides affect various population groups, including farm workers, residents in neighboring areas, consumers and wild animals [4,5]. Handling and consumption of these products are responsible for a series of conditions including acute intoxications [6], Parkinson’s disease [7], skin diseases [8], congenital malformations [9] and the onset of cancer after long periods of exposure [10]. An increase of 93% in the world’s consumption of pesticides was observed in the last two decades, while in Brazil, the largest consumer of pesticides in the world [11, 12], this increase was of 190%. New control alternatives are desired, where the new measures do not affect the development and production of the plant and present a lower risk of contamination for man and the environment [13,14].

Enzymes catalyze hundreds of successive reactions, consisting of highly coordinated processes indispensable for the maintenance of the life of an organism [15, 16]. Essential enzymes, which tend to be conserved between closely related organisms [17, 18] have been the subject of study as targets for diseases caused by a variety of organisms [19–25], including plant pathogens like *Pseudomonas syringae* [26] and *Xanthomonas* spp. [27]. Comparative genomic approaches, taking advantage of the huge amount of sequence data generated in the last decade, may contribute in several ways to the identification of key enzymes in the phytopathogens’ genomes [28, 29].

Enzyme classification follows rules defined by the International Union of Biochemistry and Molecular Biology Nomenclature Committee (NC-IUBMB), in association with the International Union of Pure and Applied Chemistry (IUPAC). A four-digit classification scheme known as the Enzyme Commission Number (EC) was proposed by this committee [30]. The first three digits are those that define the catalyzed reaction, the second and third comprise the subclasses of the reactions, and the fourth digit is a unique identifier that corresponds to the catalytic activity itself. Enzymes can also be grouped into families based on sequence similarity, and families are organized into superfamilies according to the catalytic activity [31]. Sequence motifs and domain architecture are the main criteria employed, but other characteristics can be used [32]. This diversity may result in functional overlap: these cases are known as non-homologous isofunctional enzymes (NISEs), also known as functional analogous enzymes [33, 34]. Analogous enzymes perform the same biochemical function, but have different evolutionary origins, with distinct primary structures whose differences are reflected in their tertiary structures [35]. Convergent evolution, initially thought to be a rare phenomenon in enzyme evolution, has been demonstrated for several enzymes including superoxide dismutase [36–38] and proteases [39]. Later, cases of functional analogy were found in most biochemical pathways [40–42]. Most importantly, the structural differences found between analogous enzymes from the plant and the phytopathogen, a consequence of their different evolutionary origins, may be exploited for the design of specific molecules that will interact only with the form found in the phytopathogen, leaving the plant and other important species, particularly men itself and *Apis mellifera*, one of the most important pollinators [43,44], unharmed.

Thus, the objective of this study was to develop and implement a computational approach to i) identify and validate a set of NISEs, ii) reveal a subset of essential analogous enzymes and iii) disclose a subset of specific enzymatic structures, possessed only by the pathogens. To test our approach, we studied the genomes of three plants of great economic importance and worldwide distribution, *Glycine max*, *Zea mays* and *Solanum lycopersicum*, 15 bacterial and fungal plant pathogens, the genomes of *Homo sapiens*, *Apis mellifera* and two beneficial microorganisms, *Bacillus subtilis* and *Trichoderma harzianum*.

## Material and methods

**Fig 1:**
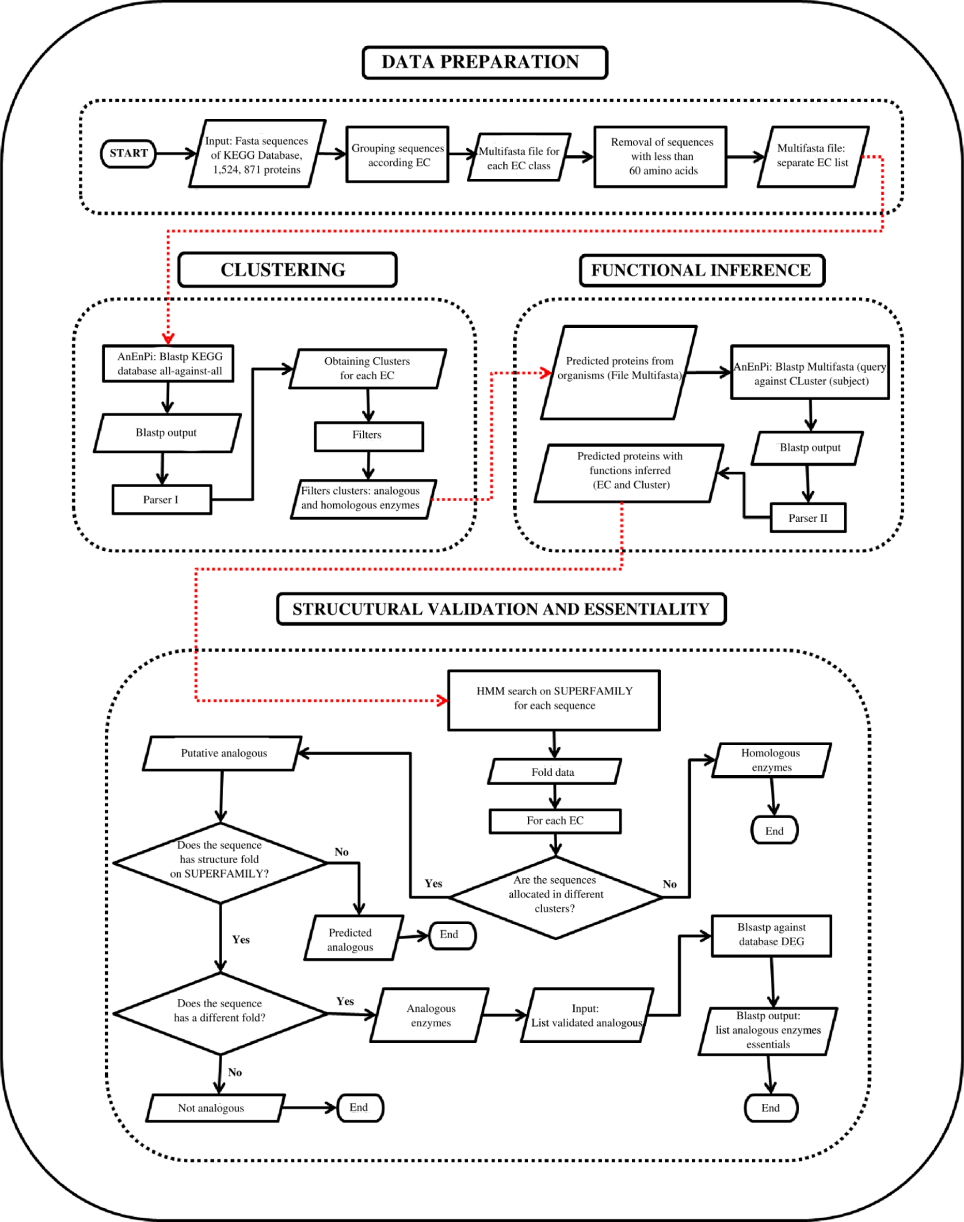
Identification of essential, non-homologous isofunctional enzymes.

The analyzes were performed in four main stages: data preparation, clustering, functional inference, structural validation, and essentiality. A flowchart of the methodology is shown in Fig 1.

### Datasets and clustering

The datasets of predicted proteins for each genome studied in this work were obtained from UniprotKB (version 2015_10 http://www.uniprot.org/) and RefSeq (Version 70, http://www.ncbi.nlm.nih.gov/). These datasets contained several proteins annotated as "uncharacterized", "hypothetical" and / or "putative". Three plant genomes were analyzed: *G*. *max*, *Z*. *mays and S*. *lycopersicum*. Pathogens were chosen according to the geographic distribution of the disease, most of them with a cosmopolitan occurrence. The pathogens analyzed comprise eleven fungal and four bacterial genomes, all pathogenic to one or more species of the plants studied. Also included were the genomes of *Homo sapiens*, *Apis mellifera* (pollinator), *Trichoderma harzianum* (soil fungus) and *Bacillus subtilis* (plant growth promoting bacteria) (Table 1).

**Table 1:** Description of the predicted proteins datasets of the organisms included in this study.

Organisms	Database	Accession NCBI	Reference	#Ptn	Unch.	Hyp.	Put.	Annot. (%)
*Glycine max*	RefSeq	NC_016088	[45]	59374	23618	__	1566	61
*Aspergillus flavus* ^1^*	RefSeq	GCA_000006275.2	[46]	13287	5380	__	__	59
*Fusarium oxysporum* ^2^*	Uniprot	GCA_000222805.1	[47]	17385	16,684	__	1	8
*Phytophthora sojae* *	RefSeq	AAQY00000000	[48]	26106	__	25279	125	2,8
*Sclerotinia sclerotiorum* *	RefSeq	AAGT00000000.1	[49]	12902	12,042	__	3	6,6
*Xanthomonas axonopodis* **	RefSeq	CP004399	[50]	4496	1413	__	35	67
*Solanum lycopersicum*	Uniprot	AEKE00000000	[51]	31683	28785	__	__	9,1
*Botrytis cinerea* *	RefSeq	NZ_AAID00000000.1	[52]	14687	__	8,696	__	40
*Fusarium oxysporum* ^3^*	Uniprot	GCA_000149955.2	[53]	15811	15,148	__	__	4,3
*Moniliophthora perniciosa* *	Uniprot	ABRE00000000	[54]	12915	12,741	__	__	1,3
*Pseudomonas syringae* **	RefSeq	NC_004578.1	[55]	5449	__	1446	__	73
*Ralstonia solanacearum* **	RefSeq	NC_003295.1	[56]	4400	696	135	1292	56
*Zea mays*	RefSeq	LPUQ00000000	[57]	59384	__	2363	2300	92
*Aspergillus flavus* ^4^*	RefSeq	GCA_000952835.1	[58]	13561	__	5423	5884	16
*Colletotrichum graminicola* *	RefSeq	ACOD00000000	[59]	11910	__	5,381	__	54
Gibberella moniliformis*	Uniprot	AAIM00000000.2	[60]	17384	13,71	__	__	21
*Exserohilum turcicum* *	RefSeq	AIHT00000000	[61]	4248	__	11159	1	3,6
*Pantoea ananatis* **	RefSeq	CP001875	[62]	4302	707	__	14	83
*Apis mellifera*	Uniprot	AADG00000000	[63]	13514	12511	__	5	7,3
*Trichoderma harzianum* *	Uniprot	MRYK00000000	[64]	11480	7704	__	3	32
Bacillus subtilis**	Uniprot	NC_000964	[65]	26433	1299	__	301	93
*Homo sapiens*	Uniprot	CM000663	[66]	63487	1338	__	1071	96

^__^No proteins in this category

* Fungi

** Bacteria

^1^ *A*. *flavus* NRRL3357

^2^ *F*. *oxysporum* Fo5176

^3^ *F*. *oxysporum* 4287

^4^ *A*. *flavus* AF70.

#Ptn., total number of proteins; Unch., uncharacterized proteins; Hyp., hypothetical proteins; Put.,putative proteins; Annot.%, annotation percentage

The complete, annotated set of enzymes was extracted from KEGG (release 73.0, January 2015) and contained 1,524,871 protein sequences, from 298 Eukaryotes, 3014 Eubacteria and 175 Archaea genomes. Sequences with less than 60 amino acids were removed. To clusterize the sequences into groups based on sequence similarity, we used the AnEnPi pipeline [67]. A similarity score with a cut-off value of 120 was used for all BLASTp pairwise comparisons since this score separates enzymes with different tertiary structures [34]. Results were parsed to obtain, for each enzymatic activity as defined by their Enzyme Commission (EC) number, files containing one or more groups of primary structures. If for a given enzymatic activity, only one group was produced at the end of the clusterization step, then all sequences would be considered homologous, and that enzymatic activity was removed from the analysis. On the other hand, if more than one group was produced, then sequences in the same group were considered homologous, with a score above 120, while sequences allocated in different groups were considered analogous (potential NISEs), with a score smaller than 120. In other words, sequences allocated in the same group have similar tertiary structures, while sequences allocated in different groups have different folding patterns, which reflects their different evolutionary origins [34, 35, 68].

### Protein function inference

The groups of homologous sequences generated after the clustering step using the KEGG dataset were used for reannotation (with the pipeline AnEnPi) of the predicted proteins from the organisms in this study, which were compared, in a pairwise manner, to each primary protein structure within each protein functional group from KEGG. For the biochemical function inference, a cutoff value of 10^−20^ was used, a highly restrictive value that gives greater reliability to the results [67, 69–71]. Sequences with scores below this threshold were removed from the analysis.

### NISEs: Identification, structural validation and essentiality

The search for cases of analogy (NISEs) between enzymes from plants and pathogens was performed through the analysis of the groups produced after the clustering step and functional inference. For this, one of the modules of AnEnPi was used together with in-house scripts to parse and filter the results. To validate the identified NISEs, that is, to verify if the enzymes found are cases of evolutionary convergence, we classified the sequences in accordance with their folds using the SUPERFAMILY database. The information in this database is based on a collection of Hidden Markov Models [72], which represent the structural domains of proteins classified by SCOP [73].

Heteromultimeric enzymes, enzymes annotated with the term "subunit" and sequences without an associated fold were excluded from the final list. Fused domains were maintained in our analysis, as in the case of the family "Dimeric alpha + beta barrel", which is an evolutionarily conserved group of protein families [73, 74]. Enzymes with the same EC number, but displaying different folds and, consequently, belonging to different superfamilies, were considered potential NISEs.

The Database of Essential Genes (DEG, 14.7, October/2016, http://www.essentialgene.org/) was used as a reference for the search for essential activities in the pathogens studied. A BLASTp search was performed between all enzymatic sequences identified as analogous against the DEG database. An e-value of 10^−5^ was used as threshold. Later, another BLASTp search was performed between all enzymatic sequences identified as analogues against the predicted proteins of organisms that should not be affected by an eventual inhibitor for the target identified in phytopathogen (*H*. *sapiens*, *A*. *mellifera*, *T*. *harzianum* and *B*. *subtilis*). An e-value of 10^−5^ was used as threshold.

## Results

### Data preparation, clustering and functional activity inference

After cleaning and preparation, the initial dataset obtained from KEGG was reduced to 1,225,682 protein sequences distributed over 3,893 enzymatic activities. After clusterization, this dataset was used for the reannotation of the predicted proteins of the plants and phytopathogens, comprising 444198 individual sequences in 2096 enzymatic activities from the three plants and their 15 pathogens. Predicted proteins from *H*. *sapiens*, *A*. *mellifera*, *T*. *harzianum* and *B*. *subtilis* were also reannotated, comprising 114914 individual sequences in 2008 enzymatic activities. Annotation quality of the downloaded sets of predicted proteins varied greatly. Before the reannotation procedure, the best annotated organism among the plants was *Z*. *mays*, with approximately 90% of their proteins characterized, while *S*. *lycopersicum* presented only 9% of its proteins annotated. Among the pathogens, *P*. *ananatis* presented 83% of its entire conceptual proteome annotated and *M*. *perniciosa* had only 1.3% of its proteins characterized. After the functional inference step, where only enzymes were reannotated, on average 15% of the proteins of each organism were associated with an enzymatic activity (data not shown).

### Potential NISEs: Identification and validation

Initially, a total of 568 cases of potential NISEs was identified, and from this set 97 cases were validated (Table 2, see S1 Table for more details). Sequences labeled with "subunit" or "chain" (324 cases), enzymes displaying the same fold (55 cases), and sequences without an associated fold in the SUPERFAMILY database (92 cases) were excluded. Cases of analogy were validated for all the pathogens studied: only one case was found for *P*. *sojae* and *S*. *sclerotiorum*, while 14 cases were found for *A*. *flavus* AF70. In total, 13 cases of analogy were found in the comparisons between *G*. *max* and its pathogens, 23 cases between *S*. *lycopersicum* and its pathogens, and 61 cases between *Z*. *mays* and its pathogens (Table 2).

**Table 2:** Number of potential, validated, specific and essential NISEs. Numbers in parenthesis indicate the number of enzymatic activities identified.

Host	Pathogens	Potential NISEs	Validated	Specific*	Essential
*G*. *max*	*A*. *flavus*^1^	25	4	3	2
	*F*. *oxysporum*^2^	21	4	4	1
	*P*. *sojae*	25	1	1	1
	*S*. *sclerotiorum*	21	1	1	0
* *	*X*. *axonopodis*	12	3	2	2
*S*. *lycopersicum*	*B*. *cinerea*	18	3	2	1
	*F*. *oysporum*^3^	30	6	5	2
	*M*. *perniciosa*	23	4	2	2
	*P*. *syringae*	38	5	4	5
* *	*R*. *solanacearum*	32	5	5	5
*Z*. *mays*	*A*. *flavus*^4^	64	14	8	7
	*C*. *graminicola*	69	13	7	9
	*E*. *turcicum*	62	12	7	9
	*G*. *moniliformis*	65	10	6	5
* *	*P*. *ananatis*	63	12	11	7
Total		568	97 (39)	68 (26)	58 (29)

* Number of pathogen’s specific tertiary structures

^1^ *A*. *flavus* NRRL3357

^2^ *F*. *oxysporum* Fo5176

^3^ *F*. *oxysporum* 4287

^4^ *A*. *flavus* AF70.

**Fig 2:**
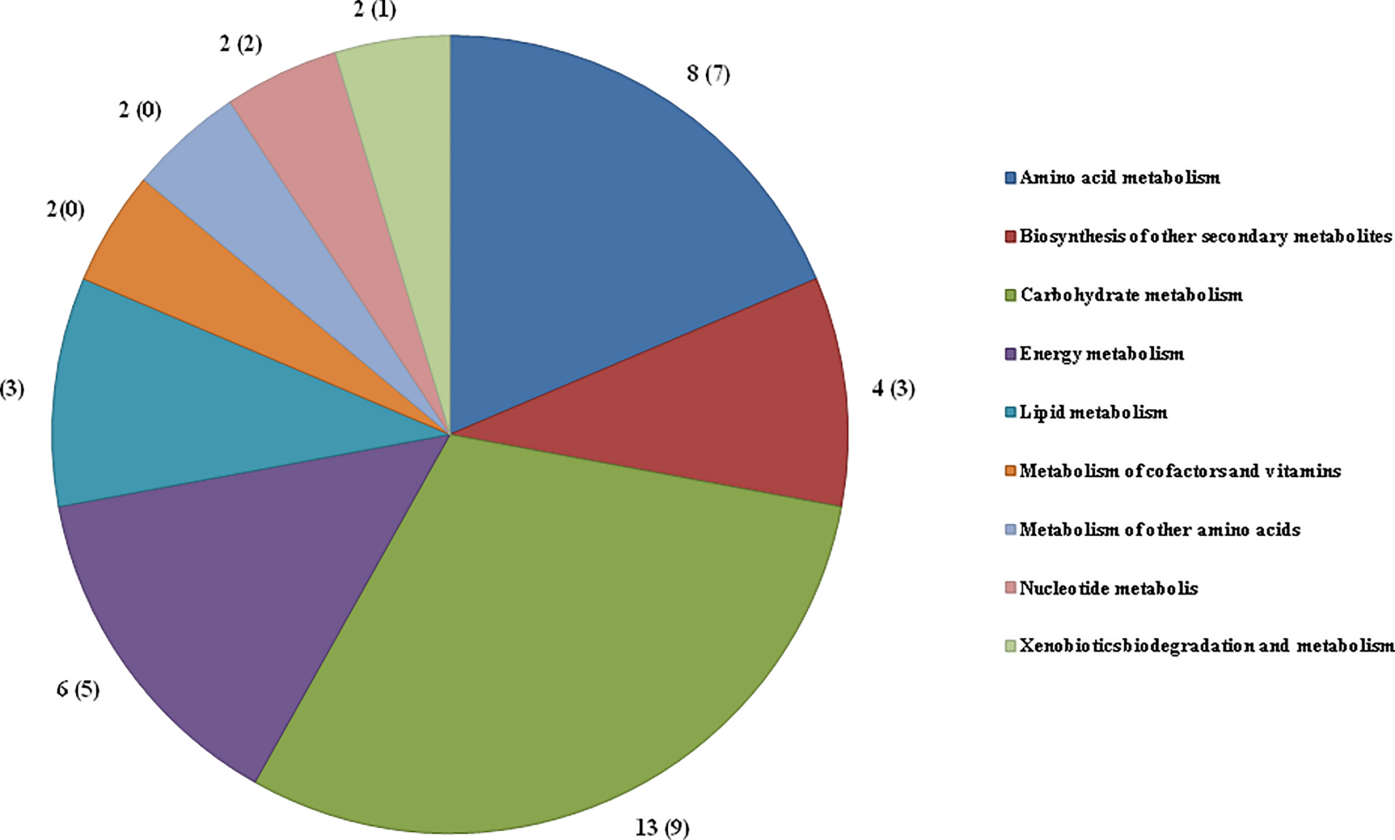
Functional classification of the validated NISEs. Numbers in parenthesis indicate the amount of essential enzymatic activities.

The validated NISEs (97 cases), comprising 39 different enzymatic activities, participate in central metabolic pathways including the carbohydrate metabolism (13 enzymatic activities), amino acid metabolism (8), energy metabolism (6), biosynthesis of secondary metabolites (4) and lipid metabolism (4). Eight enzymatic activities belong to other pathways such as xenobiotics degradation, metabolism of cofactors and vitamins, nucleotide metabolism and metabolism of other amino acids (Fig 2). It is important to remember that one enzymatic activity may participate in more than one pathway.

### Essential NISEs

After the validation step a screening for essential enzymes was performed, revealing 58 cases of analogy (Table 3), involving 29 different essential enzymatic activities, corresponding to 119 different structures, for all organisms analyzed in this study. In the carbohydrate metabolism, the most frequent case was catalase, classified as essential for three pathogens of *G*. *max* (*A*. *flavus*, *F*. *oxysporum* and *P*. *sojae*), three pathogens of *S*. *lycopersicum* (*F*. *oxysporum*, *P*. *seryngae* and *R*. *solanacearum*) and three pathogens of *Z*. *mays* (*A*. *flavus*, *E*. *turcicum* and *C*. *graminicola*). Members of the pentoses pathway, like ribose 5-phosphate isomerase, ribulose-phosphate 3-epimerase and glyoxalase I, were identified in three *Z*. *mays*’ pathogens (*A*. *flavus*, *G*. *moniliformis* and *C*. *graminicola*). Another frequent case, the enzyme cyclin-dependent kinase, was found for four of the five pathogens of *Z*. *mays* (*A*. *flavus*, *E*. *turcicum*, *C*. *graminicola* and *G*. *moniliformis*).

**Table 3:** Essential and analogous enzymes.

NISEs						Essentiality data	
Hosts	ID Sequence Host	Pathogens	ID sequence pathogens	EC number	Enzyme	ID DEG**	E-value
*G*. *max*	NP_001235974.1	*A*. *flavus*	XP_002384918.1	1.11.1.6*	Catalase	DEG10110209	2,00E-068
*G*. *max*	XP_003557098.2	*A*. *flavus*	XP_002377297.1	1.11.1.7*	Peroxidase	__	__
*G*. *max*	XP_006600684.1	*A*. *flavus*	XP_002376298.1	1.2.1.3	Aldehyde dehydrogenase (NAD+)	DEG20180006	1,00E-065
*G*. *max*	XP_006600243.1	*A*. *flavus*	XP_002382374.1	2.6.1.1	Aspartate transaminase	__	__
*G*. *max*	NP_001235974.1	*F*. *oxysporum*	9FP11|F9FP11_FUSOF	1.11.1.6*	Catalase	DEG10110209	0
*G*. *max*	XP_003555725.2	*F*. *oxysporum*	F9FYF1_FUSOF	1.15.1.1*	Superoxide dismutase	__	__
*G*. *max*	XP_006600243.1	*F*. *oxysporum*	F9G466_FUSOF	2.6.1.1	Aspartate transaminase	__	__
*G*. *max*	XP_006598804.1	*F*. *oxysporum*	F9G2J4_FUSOF	4.4.1.5	Lactoylglutathione lyase	__	__
*G*. *max*	NP_001235974.1	*P*. *sojae*	XP_009521283.1	1.11.1.6*	Catalase	DEG10110209	8,00E-115
*G*. *max*	XP_003557098.2	*S*. *sclerotiorum*	XP_001585507.1	1.11.1.7*	Peroxidase	__	__
*G*. *max*	NP_001235974.1	*X*. *axonopodis*	WP_042823856.1	1.11.1.6*	Catalase	__	__
*G*. *max*	XP_006605648.1	*X*. *axonopodis*	WP_054320474.1	1.15.1.1*	Superoxide dismutase	DEG20241649	6,00E-018
*G*. *max*	XP_006601861.1	*X*. *axonopodis*	WP_033483073.1	6.4.1.2	Acetyl-CoA carboxylase	DEG10030125	4,00E-057
*S*. *lycopersicum*	K4CN29_SOLLC	*B*. *cinerea*	XP_001560519.1	3.1.3.2	Acid phosphatase	__	__
*S*. *lycopersicum*	LGUL_SOLLC	*B*. *cinerea*	XP_001550649.1	4.4.1.5	Lactoylglutathione lyase	__	__
*S*. *lycopersicum*	P21568|CYPH_SOLLC	*B*. *cinerea*	XP_001545186.1	5.2.1.8	Peptidylprolyl isomerase	DEG20241291	1,00E-046
*S*. *lycopersicum*	K4BVX3_SOLLC	*F*. *oxysporum*	A0A0D2YKD1_FUSO4	1.11.1.6*	Catalase	DEG10110209	0
*S*. *lycopersicum*	Q7XAV2_SOLLC	*F*. *oxysporum*	A0A0D2YE80_FUSO4	1.15.1.1*	Superoxide dismutase	__	__
*S*. *lycopersicum*	K4CN29_SOLLC	*F*. *oxysporum*	A0A0D2YGA3_FUSO4	3.1.3.2	Acid phosphatase	__	__
*S*. *lycopersicum*	Q42875_SOLLC	*F*. *oxysporum*	A0A0D2XJE6_FUSO4	3.2.1.4	Cellulase	__	__
*S*. *lycopersicum*	Q8GZD8_SOLLC	*F*. *oxysporum*	A0A0D2XCV3_FUSO4	3.4.11.5	Prolyl aminopeptidase	DEG20210010	7,00E-014
*S*. *lycopersicum*	LGUL_SOLLC	*F*. *oxysporum*	A0A0D2XLV4_FUSO4	4.4.1.5	Lactoylglutathione lyase	__	__
*S*. *lycopersicum*	P15003|PER1_SOLLC	*M*. *perniciosa*	E2LX62_MONPE	1.11.1.7*	Peroxidase	__	__
*S*. *lycopersicum*	Q9FVN0|AMT13_SOLLC	*M*. *perniciosa*	E2M162_MONPE	2.7.13.3	Histidine-kinase	DEG20070330	4,00E-036
*S*. *lycopersicum*	Q8GZD8_SOLLC	*M*. *perniciosa*	E2LYM3_MONPE	3.4.11.1	Leucyl aminopeptidase	__	__
*S*. *lycopersicum*	K4CJ01_SOLLC	*M*. *perniciosa*	E2LAS1_MONPE	5.4.2.8	Phosphomannomutase	DEG20020210	5,00E-030
*S*. *lycopersicum*	K4BVX3_SOLLC	*P*. *seryngae*	NP_794283.1	1.11.1.6*	Catalase	DEG10270348	0
*S*. *lycopersicum*	P15003|PER1_SOLLC	*P*. *seryngae*	NP_794565.1	1.11.1.7*	Peroxidase	DEG10180459	4,00E-010
*S*. *lycopersicum*	K4CN29_SOLLC	*P*. *seryngae*	NP_791387.1	3.1.3.2	Acid phosphatase	DEG10290292	1,00E-084
*S*. *lycopersicum*	Q05539|CHIA_SOLLC	*P*. *seryngae*	NP_794777.1	3.2.1.14	Chitinase	DEG10250423	5,00E-019
*S*. *lycopersicum*	P21568|CYPH_SOLLC	*P*. *seryngae*	NP_791005.1	5.2.1.8	Peptidylprolyl isomerase	DEG10470303	2,00E-059
*S*. *lycopersicum*	K4BVX3_SOLLC	*R*. *solanacearum*	AGH83314.1	1.11.1.6*	Catalase	DEG10270348	0
*S*. *lycopersicum*	P15003|PER1_SOLLC	*R*. *solanacearum*	AGH86619.1	1.11.1.7*	Peroxidase	DEG10350205	2,00E-008
*S*. *lycopersicum*	Q9FVN0|AMT13_SOLLC	*R*. *solanacearum*	AGH84344.1	2.7.13.3	Histidine kinase	DEG10330275	1,00E-065
*S*. *lycopersicum*	Q05539|CHIA_SOLLC	*R*. *solanacearum*	AGH83721.1	3.2.1.14	Chitinase	DEG10260021	1,00E-017
*S*. *lycopersicum*	K4C2F1_SOLLC	*R*. *solanacearum*	AGH86735.1	4.2.1.1	Carbonic anhydrase	DEG10050308	4,00E-038
*Z*. *mays*	NP_001304298.1	*A*. *flavus*	B8NGN0_ASPFN	1.10.2.2	Quinol-cytochrome-c reductase	DEG20091193	1,00E-054
*Z*. *mays*	XP_008660914.1	*A*. *flavus*	B8NX24_ASPFN	1.11.1.6*	Catalase	DEG10110209	2,00E-068
*Z*. *mays*	XP_008664058.1	*A*. *flavus*	B8NC39_ASPFN	1.11.1.7*	Peroxidase	__	__
*Z*. *mays*	NP_001145525.1	*A*. *flavus*	B8N164_ASPFN	1.11.1.15*	Peroxiredoxin	__	__
*Z*. *mays*	XP_008664254.1	*A*. *flavus*	B8NB79_ASPFN	2.1.1.43	Histone-lysine N-methyltransferase	DEG20051547	7,00E-012
*Z*. *mays*	XP_008665261.1	*A*. *flavus*	B8N9N8_ASPFN	2.5.1.18	Glutathione transferase	__	__
*Z*. *mays*	XP_008660232.1	*A*. *flavus*	B8NQM9_ASPFN	2.6.1.1	Aspartate transaminase	__	__
*Z*. *mays*	XP_008663534.1	*A*. *flavus*	B8N9A7_ASPFN	2.7.11.22	Cyclin-dependent kinase	DEG20010254	6,00E-067
*Z*. *mays*	XP_008664470.1	*A*. *flavus*	B8NB93_ASPFN	3.1.3.2	Acid phosphatase	__	__
*Z*. *mays*	XP_008656307.1	*A*. *flavus*	B8NQT3_ASPFN	3.2.2.22	rRNA N-glycosylase	__	__
*Z*. *mays*	XP_008655471.1	*A*. *flavus*	B8NWM8_ASPFN	4.2.1.1	Carbonic anhydrase	DEG20101870	2,00E-011
*Z*. *mays*	NP_001148888.1	*A*. *flavus*	B8NT23_ASPFN	4.4.1.5	Lactoylglutathione lyase	__	__
*Z*. *mays*	NP_001149850.1	*A*. *flavus*	B8N7U5_ASPFN	5.1.3.1	Ribulose-phosphate 3-epimerase	DEG20210336	6,00E-110
*Z*. *mays*	X|P_008644870.1	*A*. *flavus*	B8NFW5_ASPFN	5.3.1.6	Ribose-5-phosphate isomerase	DEG10140248	3,00E-012
*Z*. *mays*	XP_008657765.1	*E*. *turcicum*	XP_008026270.1	1.1.1.27	L-lactate dehydrogenase	DEG20010346	1,00E-086
*Z*. *mays*	NP_001105310.2	*E*. *turcicum*	XP_008029291.1	1.11.1.6*	Catalase	DEG10110209	0
*Z*. *mays*	XP_008664058.1	*E*. *turcicum*	XP_008030871.1	1.11.1.7*	Peroxidase	DEG10400636	4,00E-080
*Z*. *mays*	NP_001145525.1	*E*. *turcicum*	XP_008025877.1	1.11.1.15*	Peroxiredoxin	__	__
*Z*. *mays*	XP_008664254.1	*E*. *turcicum*	XP_008025860.1	2.1.1.43	Histone-lysine N-methyltransferase	DEG20240496	3,00E-018
*Z*. *mays*	XP_008663534.1	*E*. *turcicum*	XP_008024068.1	2.7.11.22	Cyclin-dependent kinase	DEG20090883	2,00E-041
*Z*. *mays*	XP_008651541.1	*E*. *turcicum*	XP_008029497.1	3.1.1.31	6-phosphogluconolactonase	__	__
*Z*. *mays*	XP_008664470.1	*E*. *turcicum*	XP_008024834.1	3.1.3.2	Acid phosphatase	DEG10390008	1,00E-063
*Z*. *mays*	NP_001148888.1	*E*. *turcicum*	XP_008026072.1	4.4.1.5	Lactoylglutathione lyase	__	__
*Z*. *mays*	NP_001136955.1	*E*. *turcicum*	XP_008024266.1	4.6.1.1	Adenylate cyclase	DEG10030767	2,00E-010
*Z*. *mays*	NP_001149850.1	*E*. *turcicum*	XP_008028934.1	5.1.3.1	Ribulose-phosphate 3-epimerase	DEG20210336	1,00E-108
*Z*. *mays*	XP_008644870.1	*E*. *turcicum*	XP_008028444.1	5.3.1.6	Ribose-5-phosphate isomerase	DEG10080091	8,00E-015
*Z*. *mays*	XP_008657765.1	*C*. *graminicola*	XP_008097388.1	1.1.1.27	L-lactate dehydrogenase	DEG20010346	1,00E-091
*Z*. *mays*	XP_008660914.1	*C*. *graminicola*	XP_008098502.1	1.11.1.6*	Catalase	DEG10110209	0
*Z*. *mays*	XP_008664058.1	*C*. *graminicola*	XP_008095952.1	1.11.1.7*	Peroxidase	DEG10400636	3,00E-079
*Z*. *mays*	NP_001145525.1	*C*. *graminicola*	XP_008093145.1	1.11.1.15*	Peroxiredoxin	__	__
*Z*. *mays*	XP_008663534.1	*C*. *graminicola*	XP_008094831.1	2.7.11.22	Cyclin-dependent kinase	DEG20010254	6,00E-050
*Z*. *mays*	XP_008651541.1	*C*. *graminicola*	XP_008100128.1	3.1.1.31	6-phosphogluconolactonase	__	__
*Z*. *mays*	XP_008675577.1	*C*. *graminicola*	XP_008100081.1	3.1.1.4	Phospholipase A2	DEG20240063	2,00E-026
*Z*. *mays*	XP_008664470.1	*C*. *graminicola*	XP_008094949.1	3.1.3.2	Acid phosphatase	__	__
*Z*. *mays*	XP_008658269.1	*C*. *graminicola*	XP_008092609.1	3.1.13.4	Poly(A)-specific ribonuclease	DEG20240339	7,00E-092
*Z*. *mays*	XP_008677367.1	*C*. *graminicola*	XP_008097450.1	3.1.3.3	Phosphoserine phosphatase	DEG20211963	6,00E-052
*Z*. *mays*	NP_001148888.1	*C*. *graminicola*	XP_008096879.1	4.4.1.5	Lactoylglutathione lyase	__	__
*Z*. *mays*	NP_001149850.1	*C*. *graminicola*	XP_008091175.1	5.1.3.1	Ribulose-phosphate 3-epimerase	DEG20210336	8,00E-113
*Z*. *mays*	XP_008644870.1	*C*. *graminicola*	XP_008098210.1	5.3.1.6	Ribose-5-phosphate isomerase	DEG10080091	1,00E-015
*Z*. *mays*	NP_001145525.1	*G*. *moniliformis*	W7LPB7_GIBM7	1.11.1.15*	Peroxiredoxin	__	__
*Z*. *mays*	XP_008660232.1	*G*. *moniliformis*	W7MC41_GIBM7	2.6.1.1	Asparate transaminase	__	__
*Z*. *mays*	XP_008663534.1	*G*. *moniliformis*	W7MSL6_GIBM7	2.7.11.22	Cyclin-dependent kinase	DEG20011066	2,00E-036
*Z*. *mays*	XP_008651541.1	*G*. *moniliformis*	W7M0K8_GIBM7	3.1.1.31	6-phosphogluconolactonase	__	__
*Z*. *mays*	XP_008658269.1	*G*. *moniliformis*	W7M4G2_GIBM7	3.1.13.4	Poly(A)-specific ribonuclease	DEG20240339	1,00E-088
*Z*. *mays*	XP_008664470.1	*G*. *moniliformis*	W7NDR6_GIBM7	3.1.3.2	Acid phosphatase	DEG10390008	2,00E-013
*Z*. *mays*	XP_008655784.1	*G*. *moniliformis*	W7M5R3_GIBM7	3.2.1.4	Cellulase	__	__
*Z*. *mays*	NP_001148888.1	*G*. *moniliformis*	W7LNQ2_GIBM7	4.4.1.5	Lactoylglutathione lyase	__	__
*Z*. *mays*	NP_001136955.1	*G*. *moniliformis*	W7MFF7_GIBM7	4.6.1.1	Adenylate cyclase	DEG20090256	1,00E-090
*Z*. *mays*	NP_001149850.1	*G*. *moniliformis*	W7M917_GIBM7	5.1.3.1	Ribulose-phosphate 3-epimerase	DEG20210336	1,00E-107
*Z*. *mays*	NP_001105310.2	*P*. *ananatis*	D4GMF4_PANAM	1.11.1.6*	Catalase	__	__
*Z*. *mays*	XP_008667406.1	*P*. *ananatis*	D4GL47_PANAM	1.11.1.15*	Peroxiredoxin	DEG10030767	1,00E-006
*Z*. *mays*	XP_008672910.1	*P*. *ananatis*	D4GCI2_PANAM	1.16.3.1*	Ferroxidase	__	__
*Z*. *mays*	XP_008660532.1	*P*. *ananatis*	D4GJ68_PANAM	2.1.3.3	Ornithine carbamoyltransferase	DEG10350142	9,00E-055
*Z*. *mays*	XP_008657589.1	*P*. *ananatis*	D4GHC5_PANAM	2.3.1.51	1-acylglycerol-3-phosphate O-acyltransferase	DEG10480294	2,00E-093
*Z*. *mays*	XP_008656415.1	*P*. *ananatis*	D4GHA1_PANAM	2.7.2.3	Phosphoglycerate kinase	__	__
*Z*. *mays*	XP_008662013.1	*P*. *ananatis*	D4GMM0_PANAM	2.7.4.8	Guanylate kinase	DEG10030351	9,00E-064
*Z*. *mays*	XP_008672924.1	*P*. *ananatis*	D4GGT2_PANAM	3.1.1.5	Lysophospholipase	__	__
*Z*. *mays*	XP_008651541.1	*P*. *ananatis*	D4GFB8_PANAM	3.1.1.31	6-phosphogluconolactonase	__	__
*Z*. *mays*	XP_008650400.1	*P*. *ananatis*	D4GCE1_PANAM	3.1.3.11	Fructose-bisphosphatase	DEG10480226	2,00E-090
*Z*. *mays*	XP_008672875.1	*P*. *ananatis*	D4GMQ4_PANAM	4.2.1.96	4a-hydroxytetrahydrobiopterin dehydratase	DEG10470424	3,00E-034
*Z*. *mays*	NP_001105425.1	*P*. *ananatis*	D4GK89_PANAM	4.3.3.7	4-hydroxy-tetrahydrodipicolinate synthase	DEG10180422	1,00E-020

*Enzymes of the antioxidant system.

** Accession number in DEG.

In the amino acid metabolism, several enzymes were identified as essential and analogous, like carbonic anhydrase for *R*. *solanacearum* and. *A*. *flavus* AF70; prolyl aminopeptidase, for *F*. *oxysporum* 4287; transaminase, for *A*. *flavus* AF70, *G*. *moniliformis*, *A*. *flavus* NRRL3357 and *F*. *oxysporum* Fo5176. Chitinases were found as essential and analogous for *P*. *seryngae* and *R*. *solanacearum* (Table 3).

Analogous and essential enzymes were also found in the metabolism of lipids and biosynthesis of secondary metabolites pathways. Acetyl-CoA carboxylase was identified in. *X*. *axonopodis* and phospholipase A2 in *C*. *graminicola*. Ornithine carbamoyltransferase, identified in *P*. *ananatis*, participates in the amino acid metabolism (S2 Table). Some enzymatic activities found to be essential for some pathogens have not been identified as essential in others: these cases are represented by enzymes encoded by different genes. In this group we can cite enzymes belonging to the antioxidant system (AS), composed of enzymes involved with the detoxification of reactive oxygen species (ROS) such as catalase, peroxidase, superoxide dismutase, peroxiredoxin, among others.

### Analogous enzymes in the antioxidant system

One group of enzymes that stood out among the validated NISEs, including non-essential activities, were the enzymes that comprise the antioxidant system (AS). In all comparisons made between plants and their pathogens, except in the case of *B*. *cinerea*, for at least one of the functional activities of the antioxidant system, the host enzyme and its counterpart in the pathogen are structurally different (Table 4). In total, 27 cases of analogy were found for the antioxidant system, including catalase (CAT), peroxidase (POX), superoxide dismutase (SOD), ferroxidase (HEPH) and peroxiredoxin (PRDX). In our results, CAT was identified as an essential enzyme for 9 of the 14 pathogens studied, and POX was identified as essential in *E*. *turcicum*, *C*. *graminicola*, *P*. *seryngae* and *R*. *solanacearum*. SOD was identified as an essential enzyme for *X*. *axonopodis*. Among the pathogens analyzed, there are two species with distinct strains, *A*. *flavus* (NRRL3357, AF70) and *F*. *oxysporum* (Fo5176, 4287). No differences were observed between different lineages as in the case of *A*. *flavus* and *F*. *oxysporum*. It is important to emphasize that the AS enzymatic activities are present in all the genomes included in the present work; however, only the cases of validated NISEs have been shown, which explain gaps in the absence/presence pattern observed for HEPH, PRDX and SOD (Table 4).

**Table 4:** Alternative enzymatic forms found among the enzymes of the antioxidant system.

Organisms	Structural forms																						
	CAT					POX						SOD					HEPH			PRDX			
*G*. *max*	①*			⑤		③	⑥				⑳	①	④	⑥	⑦								
*A*. *flavus*^1^		❷						⓬															
*F*. *oxysporum*^2^		❷														⓮							
*P*. *sojae*		❷																					
*S*. *sclerotiorum*								⓬															
*X*. *axonopodis*			❸																				
*S*. *lycopersicum*	①			⑤		③	⑥					①	④	⑥	⑦								
*B*. *cinerea*																							
*F*. *oysporum*^3^		❷														⓮							
*M*. *perniciosa*								⓬															
*P*. *syringae*		❷							⓰														
*R*. *solanacearum*					❻					⓲													
*Z*. *mays*	①			⑤		③											②	⑥		①			⑩
*A*. *flavus*^4^		❷						⓬														❾	
*C*. *graminicola*		❷					❻															❾	
*E*. *turcicum*		❷					❻															❾	
*G*. *moniliformis*																						❾	
*P*. *ananatis*			❸																❼		❷		

^1^ *A*. *flavus* NRRL3357

^2^ *F*. *oxysporum* Fo5176

^3^ *F*. *oxysporum* 4287

^4^ *A*. *flavus* AF70.

*Numbers represent the groups where a sequence was located. Only validated cases of analogy are shown. Black circles indicate structural forms validated found only on the pathogen.

### Specific structural forms

After obtaining the final list of validated, essential NISEs between the plant hosts and their pathogens, a search for these enzymatic activities was performed on the predicted proteins of *H*. *sapiens*, *A*. *mellifera*, *B*. *subtilis* and *T*. *harzianum*. The objective of this comparison was to find specific structural enzymatic forms of the pathogen in the genomes of species that should not be affected by an eventual inhibitor targeting that particular structural form, mainly *H*. *sapiens* and *A*. *mellifera*. Of the 97 NISEs validated, 68 specific structural forms of the pathogen (in relation to the plant host, men and bee) were found (Table 5). They are distributed over 26 enzymatic activities (16 of them being essential). From these 68 structural forms, 39 were present in *T*. *harzianum* and 17 in *B*. *subtilis*, which is expected since these organisms belong to the same kingdoms of the phytopathogens studied in this work (Fungi and Bacteria).

**Table 5:** Phytopathogen specific enzymatic structural forms.

Comparison				Structural forms					
Plant**	Pathogen**	EC Number	ID Sequence Pathogens	Pathogens	Plant	*H*. *sapiens*	*A*. *mellifera*	*T*. *harzianum*	*B*. *subtilis*
Gm	Af	1.11.1.6‡	XP_002384918.1	1^Δ^, **2**	1, 5	1, 5	1, 5	1, 2*	1, 3, 8
Gm	Af	1.11.1.7	XP_002377297.1	3, 6, **12**	3, 6, 20	1, 3	1, 3, 6	3, 6, 12*	7
Gm	Af	2.6.1.1	XP_002382374.1	1, **5**	1	1	1	1, 5*	1
Gm	Fo	1.11.1.6‡	F9FP11_FUSOF	1, **2**	1, 5	1, 5	1, 5	1, 2*	1, 3, 8
Gm	Fo	1.15.1.1‡	F9FYF1_FUSOF	1, 4, 7, **14**	1, 4, 6, 7	1, 4, 7	1, 4, 7	1, 4, 7, 14*	1, 4
Gm	Fo	2.6.1.1	F9G466_FUSOF	1, **5**	1	1	1	1, 5*	1
Gm	Fo	4.4.1.5	F9G2J4_FUSOF	1, **3**	1, 8	1	1	1, 3*	1, 3, 6, 7, 11
Gm	Ps	1.11.1.6‡	XP_009521283.1	1, **2**	1, 5	1, 5	1, 5	1, 2*	1, 3, 8
Gm	Ss	1.11.1.7	XP_001585507.1	3, 6, **12**	3, 6, 20	1, 3	1, 3, 6	3, 6, 12*	7
Gm	Xa	1.11.1.6	WP_042823856.1	**3**	1, 5	1, 5	1, 5	1, 2	1, 3*, 8
Gm	Xa	6.4.1.2‡	WP_033483073.1	1, **6**	1	1	2	1	1, 6*
Sl	Bc	3.1.3.2	XP_001560519.1	2, 3, 7, **13**	2, 6, 9, 11	2, 4, 7	2, 4, 5, 7, 20	2, 3, 4, 5, 7, 13*	__
Sl	Bc	4.4.1.5	XP_001550649.1	1, **3**	1, 8	1	1	1, 3*	1, 3*
Sl	Fo	1.11.1.6‡	A0A0D2YKD1_FUSO4	1, **2**, 5	1, 5	1, 5	1, 5	1, 2*	1, 3, 8
Sl	Fo	1.15.1.1	A0A0D2YE80_FUSO4	1, 4, 7, **14**	1, 4, 6, 7	1, 4, 7	1, 4, 7	1, 4, 7, 14*	1, 4
Sl	Fo	3.1.3.2	A0A0D2YGA3_FUSO4	**1**, 2, 3, 4, 7, 13	2, 4, 6, 9	2, 4, 7	2, 4, 5, 7, 20	2, 3, 4, 5, 7, 13	__
Sl	Fo	3.2.1.4	A0A0D2XJE6_FUSO4	1, **6**	1	__	1	1	1, 3
Sl	Fo	4.4.1.5	A0A0D2XLV4_FUSO4	1, **3**	1, 8	1	1	1, 3*	1, 3*, 6, 7, 11
Sl	Mp	1.11.1.7	E2LX62_MONPE	6, **12**	3, 6	1, 3	1, 3, 6	3, 6, 12*	7
Sl	Mp	3.4.11.1	E2LYM3_MONPE	1, **11**	1	1	1	__	1
Sl	Psy	1.11.1.6‡	NP_794283.1	1, **2**	1, 5	1, 5	1, 5	1, 2*	1, 3, 8
Sl	Psy	1.11.1.7‡	NP_794565.1	6, **16**, 18, 19	3, 6	1, 3	1, 3, 6	3, 6, 12	7
Sl	Psy	3.1.3.2‡	NP_791387.1	**1**, 3	2, 6, 9, 11	2, 4, 7	2, 4, 5, 7, 20	1*, 3, 4, 5, 7, 13	__
Sl	Psy	3.2.1.14‡	NP_794777.1	1, **3**	1	1, 10	1, 4	1	__
Sl	Rs	1.11.1.6‡	AGH83314.1	**6**	1, 5	1, 5	1, 5	1, 2	1, 3, 8
Sl	Rs	1.11.1.7‡	AGH86619.1	6, **18**	3, 6	1, 3	1, 3, 6	3, 6, 12	7
Sl	Rs	2.7.13.3‡	AGH84344.1	1, **21**, 23, 24, 33, 36	1, 20	2, 12, 13, 20	12, 20	1	1
Sl	Rs	3.2.1.14‡	AGH83721.1	**3**	1	1, 10	1, 4	1	__
Sl	Rs	4.2.1.1‡	AGH86735.1	1, 3, **13**	1, 2, 5	1, 2	1, 2, 3	1, 2	1, 3, 5, 12
Zm	Af	1.11.1.15	|B8N164_ASPFN	1, **9**	1, 10	1	1	1, 9*	1
Zm	Af	1.11.1.6‡	B8NX24_ASPFN	1, **2**	1, 5	1, 5	1, 5	1, 2*	1, 3, 8
Zm	Af	1.11.1.7	B8NC39_ASPFN	3, 6, **12**	3	1, 3	1, 3, 6	3, 6, 12*	7
Zm	Af	2.6.1.1	B8NQM9_ASPFN	1, **5**	1	1	1	1, 5*	1
Zm	Af	3.1.3.2	B8NB93_ASPFN	2, **13**	2, 4, 6, 9	2, 4, 7	2, 4, 5, 7, 20	2, 3, 4, 5, 7, 13*	__
Zm	Af	3.2.2.22	B8NQT3_ASPFN	**5**	1, 7	__	__	__	__
Zm	Af	4.4.1.5	B8NT23_ASPFN	1, **3**	1, 8	1	1	1, 3*	1, 3*, 6, 7, 11
Zm	Af	5.3.1.6‡	B8NFW5_ASPFN	1, **2**	1	1	1	1, 2*	__
Zm	Cg	1.1.1.27‡	XP_008100733.1	**2**, 12	1, 12	1, 12	1, 12	1, 2*, 12	1, 11
Zm	Cg	1.11.1.15	XP_008093145.1	1, **9**	1, 10	1	1	1, 9*	1, 9*
Zm	Cg	1.11.1.6‡	XP_008098502.1	1, **2**, 5	1, 5	1, 5	1, 5	1, 2*	1, 3, 8
Zm	Cg	3.1.1.31	XP_008100128.1	1, **2**	1	1, 4	1	1, 2*	2*
Zm	Cg	3.1.3.2	XP_008094949.1	2, 3, 5, **13**	2, 4, 6, 9	2, 4, 7	2, 4, 5, 7, 20	2, 3, 4, 5, 7, 13*	__
Zm	Cg	4.4.1.5	XP_008096879.1	1, **3**	1, 8	1	1	1, 3*	1, 3*, 6, 7, 11
Zm	Cg	5.3.1.6‡	XP_008098210.1	1, **2**	1	1	1	1, 2*	2*
Zm	Et	1.11.1.15	XP_008025877.1	1, **9**	1, 10	1	1	1, 9*	1
Zm	Et	1.11.1.6‡	XP_008029291.1	1, **2,** 5	1, 5	1, 5	1, 5	1, 2*	1, 3, 8
Zm	Et	3.1.1.31	XP_008029497.1	1, **2**	1	1, 4	2	1, 2*	2*
Zm	Et	3.1.3.2‡	XP_008024834.1	**1**, 2, 3, 13	2, 4, 6, 9	2, 4, 7	2, 4, 5, 7, 20	1*, 3, 4, 5, 7, 13	__
Zm	Et	4.4.1.5	XP_008026072.1	1, **3**	1, 8	1	1	1, 3*	1, 3*, 6, 7, 11
Zm	Et	4.6.1.1‡	XP_008024266.1	2, 8, **10**	2, 17, 18	2, 8	2, 6, 8, 13	2, 8	4
Zm	Et	5.3.1.6‡	XP_008028444.1	1, **2**	1	1	1	1, 2*	__
Zm	Gm	1.11.1.15	W7LPB7_GIBM7	1, 2, **9**	1, 10	1	1	1, 9*	1
Zm	Gm	2.6.1.1	W7MC41_GIBM7	1, **5**	1	1	1	1, 5*	1
Zm	Gm	3.1.1.31	W7M0K8_GIBM7	1, **2**	1	1, 4	1	1	2*
Zm	Gm	3.1.3.2‡	W7NDR6_GIBM7	1, 2, 3, 7, **13**	2, 4, 6, 9	2, 4, 7	2, 4, 5, 7, 20	2	__
Zm	Gm	3.2.1.4	W7M5R3_GIBM7	1, **6**	1	__	1	1	1, 3
Zm	Gm	4.4.1.5	W7LNQ2_GIBM7	1, **3**	1, 8	1	1	1	1, 3*, 6, 7, 11
Zm	Pa	1.11.1.15‡	D4GL47_PANAM	1, **2**	1, 10	1	1	1	1
Zm	Pa	1.11.1.6	D4GMF4_PANAM	**3**, 5, 6	1, 5	1, 5	1, 5	1	1, 3*, 8
Zm	Pa	1.16.3.1	D4GCI2_PANAM	2, **7**	2, 6	2, 4, 6	2, 6	6	1
Zm	Pa	2.1.3.3‡	D4GJ68_PANAM	**2**	1, 10	1, 10	10	1	1, 10
Zm	Pa	2.7.2.3	D4GHA1_PANAM	**3**	1	1	1	1	1, 3*, 9
Zm	Pa	2.7.4.8	D4GMM0_PANAM	1, **4,** 7	1	1	1	1	1, 7
Zm	Pa	3.1.1.31	D4GFB8_PANAM	**2**, 6	1	1, 4	1	1	2*
Zm	Pa	3.1.1.5	D4GGT2_PANAM	2, **5**	6, 7, 18	1, 4, 6, 7, 9, 10, 17, 18	1, 6, 7, 9, 17, 18	1	__
Zm	Pa	3.1.3.11‡	D4GCE1_PANAM	**10**, 12	1	1, 8	1	1	3, 11
Zm	Pa	4.2.1.96‡	D4GMQ4_PANAM	**2**	1	1	1	1	2*
Zm	Pa	4.3.3.7‡	D4GK89_PANAM	1, **2**	1	__	__	__	1, 4

**Gm: *G*. *Max*, Af: *A*. *flavus*, Fo: *F*. *oxsyporum*, Ps: *P*. *sojae*, Ss: *S*. *sclerotiorum*, Xa: *X*. *axonopodis*, Sl: *S*. *lycopersicum*, Rs: *R*. *solanacearum*, Psy: *P*. *syringae*, Mp: *M*. *perniciosa*. Bc: *B*. *cinerea*, Zm: *Z*. *mays*, Pa: *P*. *ananatis*, Gm: *G*. *moniliformis*, Et: *E*. *turcicum*, Gg: *C*. *graminicola*.

^Δ^ Numbers represent the different structures. Numbers in bold are the specific phytopathogen enzymatic structural forms.

‡ Essential enzymes.

^__^ Enzymatic activity not found.

*Structural form homologous to the pathogen.

## Discussion

The correct description of the analogous enzymes is important for the practical tasks of metabolic reconstruction and enzymatic nomenclature. In addition to this practical importance, these enzymes represent important evolutionary phenomenon, existence shows that for various biochemical problems, evolutionarily independent solutions may appear [35]. The main works on the practical application of analogous enzymes describes studies of metabolic pathways and inhibitory targets for human pathogens [42, 69–70]. In the case of our study, we sought a practical application, focused on the solution of an agronomic problem.

Essential enzymes are one of the primary targets for the development of inhibitors of any kind; however, species that share essential enzymatic functions may inadvertently be affected by products developed with other applications in mind [75]. Pesticides are commonly targeted at these functions, and their damaging effects on several species including man himself and several vital species such as pollinators and beneficial microorganisms are reason for great concern [76–78]. In fact, it is estimated that approximately 35% of the crops are dependent on pollinators for sexual reproduction, and pesticides are the main factor contributing to the current decrease of the pollinator population [44, 79].

Through the joint use of primary structure data, tertiary structure data and essentiality data, beginning with 444198 individual sequences, comprising 2096 enzymatic activities in 3 plants and 15 phytopathogens, we have disclosed a subset of analogous sequences in 29 essential enzymatic activities present both in the plant and the pathogen. These belong to several components of the central metabolism of plant and pathogens, being involved in the carbohydrate metabolism, the metabolism of amino acids, the detoxification of reactive oxygen species and others, thus offering several opportunities as targets.

Interestingly, the subset of non-essential NISEs contains several enzymes important in the context of host-pathogen interactions, such as cellulases, chitinases, glutathione transferase and lysophospholipase. Blocking or inhibiting these enzymes would, in principle, decrease virulence and / or delay the defense mechanisms of the pathogen [80, 81]. Inhibition of cellulases and chitinases has also been proposed as a strategy for the development of new antifungal drugs for aspergillosis in humans [22]. Glutathione transferase play an essential role in the protection of necrotrophic fungi against toxic metabolites derived from plants and reactive oxygen species [82], while lysophospholipase has been implicated with virulence in *Cryptococcus neoformans* [83].

Some of the diversity found for the enzymes of the antioxidant system, both in terms of enzymatic activities and in structural forms, may be explained by evolutionary pressures: during the co-evolution between plants and their pathogens, it is likely that different antioxidant enzymes of plants have adapted to overcome the pathogen virulence mechanisms [84, 85]. The role of these enzymes in mechanisms of virulence, susceptibility to infections, development of drug targets and evaluation of pesticide effects has been studied for SOD [86–90], CAT [91–94] and POX [95].

Essential enzymes from the central metabolism have also been studied as potential drug targets in several organisms. Glucose-6-phosphate isomerase has been studied as a target for infections caused by *Plasmodium falciparum* [96], *Trypanossoma* spp [97], *Toxoplasma gondii* [98], and *Leishamania ssp* [99], acetyl-CoA carboxylase for *L*. *major* [100, 101], and ribose 5-phosphate isomerase in other organisms [102]. Deletion of these genes usually results in a severe reduction in growth rates and virulence [103–105], and they have been studied as drug targets in other organisms [106–109].

Eighteen of the 29 enzymatic activities identified in this study as analogous and essential were identified in databases of drug targets such as TDR Drug Targets (http://tdrtargets.org/), DrugBank (https://www.drugbank.ca/) and Potential Drug Target Database (http://www.dddc.ac.cn/pdtd/), meaning they are being studied or employed as a drug target for at least one pathogen. Among them we can mention enzymes from the carbohydrate and amino acids metabolism such as lactoylglutathione lyase, acetyl-CoA carboxylase, carbonic anhydrase, and enzymes of the AS like catalase, peroxidase, peroxiredoxin and superoxide dismutase. Since these enzymatic activities present multiple tertiary structures, we are not able to tell, from this data, which one is under study; nonetheless, these findings give indirect support to our analyzes, corroborating the idea that essential enzymes with specific structural forms have great potential as drug targets as described in our study. Improvements in the annotation of genes and their products, and a better experimental characterization of enzymatic activities, would allow the use of less-stringent criteria in our procedures, mainly in data cleaning and filtering, but also in clustering and structural validation, increasing the number of essential and analogous enzymes that could be further studied as potential drug targets.

## Conclusions

The approach employed in this study enabled the elaboration of lists of essential and analogous enzymes, most belonging to the central metabolism and/or involved in host-pathogen interactions, with potential to be a drug target. These enzymes provide an opportunity for the discovery of targets with considerable structural differences over their counterpart in beneficial organisms such as pollinators. Inclusion of structural data allows the disclosure of specific structural forms, facilitating the development of environment-friendly enzyme inhibitors, which may be of great importance for agricultural use.

## Supporting information

S1 Table Non-homologous isofunctional enzymes found in this study. (XLS)

S2 Table Distribution of metabolic pathways in essential analogous enzymes. (XLS)
